# Cross-sectional, multicenter study comparing sex differences in patients undergoing endovascular repair of infrarenal abdominal aortic aneurysms. Results from the São Paulo State University Provincial Hospitals Registry (RHEUNI)

**DOI:** 10.1590/1677-5449.202400042

**Published:** 2024-10-28

**Authors:** Regina Moura, Edwaldo Edner Joviliano, Ana Terezinha Guillaumon, Selma Regina de Oliveira Raymundo, Ludwig Hafner, Marcone Lima Sobreira, Marcelo José de Almeida, Daniel Gustavo Miquelin, Martin Geiger, Winston Bonetti Yoshida

**Affiliations:** 1 Universidade Estadual Paulista – UNESP, Faculdade de Medicina de Botucatu, Botucatu, SP, Brasil.; 2 Universidade de São Paulo – USP, Faculdade de Medicina de Ribeirão Preto, Ribeirão Preto, SP, Brasil.; 3 Universidade Estadual de Campinas – UNICAMP, Faculdade de Ciências Médicas – FCM, Campinas, SP, Brasil.; 4 Faculdade de Medicina de São José do Rio Preto – FAMERP, São José do Rio Preto, SP, Brasil.; 5 Faculdade de Medicina de Marília – FAMEMA, Marília, SP, Brasil.

**Keywords:** abdominal aortic aneurysm, endovascular procedures, postoperative complications, sex distribution studies, mortality

## Abstract

**Background:**

Abdominal aortic aneurysms (AAA) are 4-6 times more frequent among men than among women, but prognosis tends to be worse in women.

**Objective:**

To compare endovascular procedures to repair infrarenal AAA in men and women, using data from a prospective registry.

**Methods:**

This registry collected data from five university hospitals in the state of São Paulo (Brazil) from 2012 to 2022. A cross-sectional study was conducted compiling demographic data, anatomic variables (aneurysm diameter, short neck, angulated neck, calcified neck, or thrombosed neck; distal neck < 1.5 cm, and tortuous, abnormal iliac arteries), complications (leaks, conversions, and patency or stenosis of branches) and renal failure and mortality at 30 days. The chi-square test and Student’s *t* test were applied with a 5% significance level. The study was approved by the Ethics Committee (process 4040-2011).

**Results:**

A total of 152 (15.9%) of the patients were women and 799 (84.0%) were men (p < 0.05). The majority were white (80.2% of the women and 87.4% of the men). Diabetes and hypertension were significantly more frequent among the women. The most prevalent shape was fusiform, particularly among the women (95.39% vs. 89.86% among men). Mean diameter was smaller among the women (5.96 cm vs. 6.49 cm; p = 0.0056). The iliac arteries were less often involved among the women (89.40% vs. 73.58%; p = 0.00001). Blood loss was greater in the men (321.40 ml vs. 168.84 ml among the women; p < 0.05). Operative mortality was similar in both sexes, but obstructions were more frequent among the women (15.2% vs. 13.51%; p = 0.017296).

**Conclusions:**

Aneurysmal diameter was smaller and obstructive complications were more frequent among women.

## INTRODUCTION

Abdominal aortic aneurysms (AAA) are around 4-6 times more frequent among men. However, AAA tend to appear later among women and their incidence increases with age.^1^ Previous reports show that the risk of rupture of these aneurysms is four times greater among women.^2,3^ A systematic review of nine studies, with 52,018 men and 11,076 women, found 30 day-mortality rates for endovascular aneurysm repair (EVAR) of 1.4% in men and 2.3% in women. Results for open surgery were worse, with 2.8% mortality for men and 5.4% for women.^4^ Sidloff et al. analyzed data from 23,245 patients, 13% of whom were women, and found 30 day-mortality after EVAR of 0.7% in men and 1.8% in women, according to the UK National Vascular Registry (2010-2014).^5^ The Swedish national registry (n = 32,393) confirms this finding.^6^ Moreover, women and minority ethnic groups tend to exhibit worse results^7^ for both treatment methods,^8,9^ with lower female mortality for EVAR than for open surgery.^9^ Although women suffer fewer comorbidities than men associated with surgical AAA repairs, operative mortality rates were higher.^10,11^ Despite the many indicators of worse prognosis among women with AAA, there are few previous studies of AAA repair in women and the results are heterogeneous.^12^

The authors are not aware of results from AAA registries from Brazil or Latin America. As such, the objectives of the present study were to assess possible sex differences in the incidence and complications of EVAR among patients who received surgical treatment at university centers in provincial São Paulo state (which are affiliated to the São Paulo State University Provincial Hospitals Registry [RHEUNI]) over a 10 year period.

## METHODS

### Study design and population

A prospective observational study was conducted of patients with AAA who underwent endovascular treatment at five public university hospitals in provincial São Paulo (which are affiliated to the RHEUNI) from 2012 to 2022. The project was approved by the Ethics Committee (process 4040-2011). All cases on the registry were included consecutively. The decision to employ endovascular procedures was taken by the specialist surgeons at the respective institutions, all board certified by the Brazilian Society of Angiology and Vascular Surgery (SBACV).

The inclusion criteria were all patients who sought care at university centers with a diagnosis of AAA and indications for elective surgery and signed the free and informed consent form. Cases treated for ruptured aneurysms and with open surgery were excluded. The indication for surgery was patients with a largest aneurysm diameter greater than or equal to 5.0 cm, regardless of sex.

### Data collection

Tables were populated with demographic data on the cases (age, sex, ethnicity), risk factors (diabetes, hypertension, dyslipidemia, smoking, renal failure, cerebrovascular disease, coronary disease), comorbidities, symptoms, urgency, etiology, anatomic aspects (access, fusiform, saccular, diameter according to tomography, proximal and distal necks, iliac arteries), surgical aspects (route of access, need for sealing, operative time, length of hospital stay) and complications (conversion, obstructions, types of leaks, additional surgery, blood loss, and death within 30 days).

### Responsibilities of the team

Data were collected from the care routines of each institution and made accessible on-line to all members of the research team participating in the project. Every 2 months, these team members held meetings in person to discuss partial data and, at the end of the defined period, a final meeting was convened to discuss the full results and write up the manuscript.

### Statistical analysis

Continuous data were expressed as means and standard deviations and compared with Student’s *t* test for unpaired samples. Categorical data were compared using Fisher’s exact test and confidence intervals were calculated. The significance level was set at 5%. Epi-Info 7 was used for analyses.

## RESULTS

From 2012 to 2022, a total of 951 consecutive cases were registered, 152 of whom were women (15.9%) and 799 of whom were men (84.0%). During the COVID-19 pandemic, both collection and registration of data were suspended. All cases were followed up for a minimum of 30 days. The demographic data are shown in Table 1. Table 2 lists the risk factors. Table 3 shows anatomic data. Table 4 presents the surgical data and data on complications.

**Table 1:** Demographic data of registry patients, with symptoms and etiology, by sex. Frequencies as percentages.

	Characteristic	Female	Male	P value
Demographic data	Mean age (years)	70.5	71.5	0.1279
	Sex	15.98%	84.02%	< 0.05*
	White ethnicity	80.26%	87.36%	0.5214
Symptoms	Asymptomatic	57.89%	65.83%	0.06075
Etiology	Degenerative	93.42%	95.11%	0.385435
	Inflammatory	0.00%	0.26%	-

* significant difference.

**Table 2:** Risk factors of registry patients, by sex. Frequencies as percentages.

Risk factor	Female n = 152	Male n = 799	P value
Diabetes	19.7%	19.7%	0.991425
Hypertension	90.7%	44,4%	0.007463*
Dyslipidemia	45.4%	33.1%	0.003793*
Smoking	51.9%	65.3%	0.001748*
Renal failure	7.23%	7.63%	0.855092
Coronary artery disease	25.0%	20.1%	0.177873
Cerebrovascular disease	7.23%	5.9%	0.52242

* significant difference.

M = male; F = female.

**Table 3:** Anatomic aspects of the AAA, by sex. Frequencies as percentages.

Type	Female	Male	P value
Fusiform	95.39%	89.86%	0.03066*
Saccular	3.94%	8.26%	0.065407
Indefinite	0.00%	1.25%	-
Mean diameter (cm)	5.960	6.496	0.0056*
Normal neck (> 15mm)	86.18%	81.22%	0.144599
Conical neck	7.23%	5.75%	0.481166
Short proximal neck	11.18%	15.26%	0.191312
Angulated proximal neck	11.84%	8.76%	0.229527
Thrombosed neck	5.92%	4.75%	0.543545
Calcified neck	8.55%	5.38%	0.127967
Distal neck < 15 mm	0.65%	0.12%	0.188789
Preserved iliac arteries	89.40%	73.58%	0.00003*

**Table 4:** Surgical complications of AAA, by sex. Frequencies as percentages.

Complication	Female	Male	P value
Conversion	0.65%	1.00%	0.688597
Stenosis	3.28%	3.75%	0.780068
Obstructions	15.2%	13.51%	0.017396*
No leaks	81.57%	81.72%	0.965447
Type I leak	3.94%	6.38%	0.246235
Type II leak	4.60%	9.13%	0.065072
Type III leak	0.0%	0.0%	-
Type IV leak	1.97%	2.13%	0.700064
Blood loss	168.84 ml	321.40 ml	p < 0.05*
Time in ICU (hours)			p = 0.1539
Death	4.60%	4.88%	0.884482

* significant difference.

## DISCUSSION

The authors are not aware of any prospective multicenter studies of registries of AAA cases in Brazil or Latin America. The registry analyzed here was created and maintained with their own resources by a group of faculty and vascular surgeons at public universities in provincial São Paulo (affiliated to the RHEUNI group). This required an organized system for collecting information using an on-line computational system accessible to all project investigators, with a dedicated, reliable, and dynamic server and tools for statistical calculations.^13^ However, privacy and confidentiality of patient data were maintained, and there was no way of identifying patients. Regular team meetings were convened to adjudicate on data collected and partial analyses. Although they have their limitations, the importance of registries of cases is founded on analysis of specific results related to a given disease in the population.^14^

Existing international AAA registries showed evidence of certain differences in behavior according to sex.^15,16^

In a study by Deery et al.,^15^ based on Medicare data, 87% of 6,611 patients (19% women) were operated for AAA with EVAR (83% of the women and 88% of the men). Women were older (76 vs. 73 years), had smaller aneurysms (5.4 vs. 5.5 cm, p < 0.001), and had a higher prevalence of chronic obstructive pulmonary disease (22% vs. 17%, p = 0.001). Additionally, women had longer operative times, and had associated renal and peripheral vascular problems. Mortality was higher among the women (odds ratio [OR], 1.7; 95% confidence interval, 1.1-2.6; p = 0.02) and rate of complications was also higher among females (OR, 1,4; IC, 1.1-1.7; p < 0.01).

The compulsory Dutch registry (Dutch Surgical Aneurysm Audit [DSAA])^16^ included 1,561 ruptured aneurysms and 7,063 elective aneurysm repairs (13.7% women). Women were older, had significantly smaller aortic diameters at the time of rupture and had greater 30 day-mortality after emergency repair. Open repair was associated with double the mortality among women.

Among the 32,393 cases of intact AAA on the Swedish registry (aortic diameter > 3.0 cm), around 20,000 (60%) were not treated.^6^ The proportions of men and women were similar, but the frequency of rupture within 5 years was higher among the women (9.7% among the women vs. 6.9% among the men, p < 0.001). Within 5 years, 56.5% of the women and 50.4 of the men had died (p < 0.001). Rupture was the third most common cause of death (11.9% among the women and 8.7% among the men; p < 0.001). The authors concluded that an improved surveillance program should be implemented for women with AAA.

In a registry of 9,675 repairs not compliant with Society for Vascular Surgery clinical practice guidelines, results for mortality and reintervention were worse among the women.^17^

In addition to the registries, four recent systematic reviews also showed that, regardless of whether they were treated for AAA by open or endovascular surgery, women had higher mortality^18-20^ and greater frequency of ruptured aneurysms,^18^ postoperative complications,^20^ and transfusions, in addition to pulmonary and intestinal complications.^19^ A systematic review by Patel et al.^8^ observed considerable sex and ethnicity-related disparities in recruitment and outcomes in studies reporting EVAR results, although prior reports already showed evidence of these differences. Exclusively in relation to endovascular surgery for AAA,^21,22^ mortality among women was greater and reinterventions^21^ and hostile anatomy were more frequent.^22^

In response to these differences, it has been proposed that AAA in women should be operated on at smaller diameters than those established for men.^2,3,23-27^

In the current patient sample, the frequency of AAA was lower among women and ages were similar across the sexes. White ethnicity predominated equally among women and men. Clinical presentation, etiology, type of proximal and distal necks, and complicated necks were also similar across the sexes (Tables 1 and 3). Fusiform morphology was more common among women and iliac arteries were significantly more often preserved among women (Table 3). However, rates of obstruction were significantly higher among the women, possibly because of the relationship between large caliber devices and smaller arteries among the women. These findings partially corroborate the conclusions of previous analyses of AAA registries comparing men and women,^8,15,16,28^ identifying some worse outcomes among females. In contrast to many other studies, operative mortality was similar in both sexes in the present sample.

Certain limitations of the present study should be mentioned. The significantly smaller frequency of female cases in the sample impaired comparisons, preventing extrapolation to the universe of cases. Additionally, surgical indications and criteria were scrutinized by each center’s team in isolation, which could have introduced selection bias. While all the participating centers were universities and were aware of the guidelines, disparate conduct may have occurred. Finally, follow-up of cases was short (30 days), preventing analysis of morbidity and mortality over the long term. The strengths of the study lie in the prospective and consecutive registry, the sample of the Brazilian population treated by the country’s Unified Health System (Sistema Único de Saúde), and data collection using a standard protocol for all participants, which reduced loss of data, as tends to occur in retrospective studies.

In conclusion, despite the smaller diameter and greater frequency of preserved iliac arteries among the women, obstructive complications were more common among women who underwent endovascular treatment. In order to reduce complications among women, the literature suggests using lower profile endoprostheses with greater flexibility and careful deployment.
