# Supplementary material for: Cross-sectional, multicenter study comparing sex differences in patients undergoing endovascular repair of infrarenal abdominal aortic aneurysms. Results from the São Paulo State University Provincial Hospitals Registry (RHEUNI)
Source: J Vasc Bras. 2024 Oct 28;23:e20240004. doi: 10.1590/1677-5449.202400042 (PMC11529998; doi:10.1590/1677-5449.202400042)
Supplement: Translation

# Translation: Estudo transversal multicêntrico comparando o gênero no reparo endovascular de aneurismas de aorta abdominal infrarrenal. Resultados do Registro dos Hospitais Estaduais Universitários do Interior de São Paulo (RHEUNI)

**DOI:** 10.1590/1677-5449.202400041

**Authors:** Regina Moura, Edwaldo Edner Joviliano, Ana Terezinha Guillaumon, Selma Regina de Oliveira Raymundo, Ludwig Hafner, Marcone Lima Sobreira, Marcelo José de Almeida, Daniel Gustavo Miquelin, Martin Geiger, Winston Bonetti Yoshida

**Affiliations:** 1 Universidade Estadual Paulista – UNESP, Faculdade de Medicina de Botucatu, Botucatu, SP, Brasil.; 2 Universidade de São Paulo – USP, Faculdade de Medicina de Ribeirão Preto, Ribeirão Preto, SP, Brasil.; 3 Universidade Estadual de Campinas – UNICAMP, Faculdade de Ciências Médicas – FCM, Campinas, SP, Brasil.; 4 Faculdade de Medicina de São José do Rio Preto – FAMERP, São José do Rio Preto, SP, Brasil.; 5 Faculdade de Medicina de Marília – FAMEMA, Marília, SP, Brasil.

**Keywords:** aneurisma de aorta abdominal, procedimentos endovasculares, complicações pós-operatórias, estudos de gênero, mortalidade

## Abstract

**Contexto:**

O aneurisma da aorta abdominal (AAA) afeta 4-6 vezes mais frequentemente os homens do que as mulheres, porém nas mulheres o prognóstico costuma ser pior.

**Objetivo:**

Comparar os procedimentos endovasculares para correção de AAA infrarrenal em homens e mulheres, usando dados de um registro prospectivo.

**Métodos:**

Esse registro foi feito em cinco hospitais universitários do estado de São Paulo entre 2012 e 2022. Foi realizado um estudo transversal, compilando dados demográficos, anatômicos (diâmetro do aneurisma, colo curto, angulado, calcificado ou com trombo; colo distal < 1,5 cm e artérias ilíacas tortuosas, alteradas), complicações (vazamentos, conversões, perviedade ou estenoses de ramos) e insuficiência renal e mortalidade em 30 dias. Foram utilizados o teste de qui-quadrado e teste *t* de Student, com significância de 5%. O estudo foi aprovado pelo Comitê de Ética (processo 4040-2011).

**Resultados:**

Um total de 152 (15,9%) dos pacientes eram mulheres e 799 (84,0%) eram homens (p < 0,05). A maior parte eram brancos (80,2% das mulheres e 87,4% dos homens). Diabetes e hipertensão foram significativamente mais frequentes nas mulheres. O formato foi fusiforme, principalmente nas mulheres (95,39% *versus* 89,86% nos homens). O diâmetro médio foi menor nas mulheres (5,96 cm *versus* 6,49 cm; p = 0,0056). As artérias ilíacas foram menos envolvidas nas mulheres (89,40% *versus* 73,58%; p = 0,00001). A perda sanguínea foi maior nos homens (321,40 ml *versus* 168,84 ml nas mulheres; p < 0,05). A mortalidade operatória foi similar entre os sexos, mas as obstruções foram mais frequentes nas mulheres (15,2% *versus* 13,51%; p = 0,017296).

**Conclusões:**

Nas mulheres, o diâmetro aneurismático foi menor e as complicações obstrutivas foram mais frequentes.

## INTRODUÇÃO

O aneurisma da aorta abdominal (AAA) é cerca de 4-6 vezes mais frequente nos homens. Entretanto, o AAA costuma aparecer mais tardiamente nas mulheres, e a sua incidência cresce com a idade^1^ . Registros prévios mostraram risco de ruptura do aneurisma quatro vezes maior nas mulheres^2,3^ . Uma revisão sistemática reunindo nove estudos, com 52.018 homens e 11.076 mulheres, encontrou uma mortalidade de 30 dias com o reparo endovascular de aneurisma (EVAR, de *endovascular aneurysm repair*) de 1,4% em homens e 2,3% mulheres. Os resultados foram piores na cirurgia aberta, com mortalidade de 2,8% para homens e 5,4% para mulheres^4^ . Sidloff et al. avaliaram 23.245 pacientes, dos quais 13% eram mulheres, e encontraram uma mortalidade de 30 dias após EVAR em homens de 0,7%, e de 1,8% em mulheres, de acordo com o UK National Vascular Registry (2010-2014)^5^ . O registro nacional sueco (n = 32.393) corrobora esse achado^6^ . Além disso, mulheres e grupos étnicos minoritários tendem a apresentar piores resultados^7^ em ambos os métodos de tratamento^8,9^ , sendo a mortalidade feminina menor no EVAR do que na cirurgia aberta^9^ . Embora as mulheres tenham menos comorbidades do que os homens nas correções cirúrgicas nos AAA, a taxa de mortalidade operatória nelas foi maior^10,11^ . Apesar de muitos indícios do pior prognóstico em mulheres com AAA, os estudos prévios sobre reparo em mulheres parecem ser poucos e com resultados heterogêneos^12^ .

Os autores desconhecem resultados de registros de AAA feitos no Brasil ou na América Latina. Sendo assim, os objetivos do presente trabalho foram avaliar possíveis diferenças de gênero na incidência e complicações do EVAR em pacientes que procuraram os serviços universitários do interior de São Paulo (que fazem parte do Registro dos Hospitais Estaduais Universitários do Interior de São Paulo [RHEUNI]) para tratamento cirúrgico em 10 anos.

## MÉTODOS

### Desenho do estudo e população

Foi feito um estudo observacional prospectivo de pacientes com AAA que foram submetidos a tratamento endovascular em cinco hospitais universitários públicos do interior de São Paulo (que fazem parte do RHEUNI) entre 2012 e 2022. Este projeto foi aprovado pelo Comitê de Ética (processo 4040-2011). Todos os casos do Registro foram incluídos de forma consecutiva. As indicações para os procedimentos endovasculares ficaram a critério de cirurgiões especialistas das respectivas instituições, todos certificados pela Sociedade Brasileira de Angiologia e Cirurgia Vascular (SBACV).

Os critérios de inclusão foram todos pacientes que procuraram esses serviços universitários em suas cidades sede, com diagnóstico de AAA e indicação de cirurgia eletiva, e que assinaram o Termo de Consentimento Livre e Esclarecido. Foram excluídos os casos tratados por ruptura de aneurisma e casos de cirurgia aberta. As indicações cirúrgicas recaíram para pacientes com maior diâmetro do aneurisma igual ou superior a 5,0 cm, incluindo as mulheres.

### Coleta de dados

Foram tabulados dados demográficos dos casos (idade, sexo, etnia), fatores de risco (diabetes, hipertensão, dislipidemia, tabagismo, insuficiência renal, doença cérebro-vascular, doença coronariana), comorbidades, sintomas, urgência, etiologia, aspectos anatômicos (acesso, fusiforme, sacular, diâmetro pela tomografia, colos proximal e distal, artérias ilíacas), aspectos cirúrgicos (via de acesso, necessidade de selamento, tempo cirúrgico, tempo de internação) e complicações (conversão, obstruções, tipo de vazamentos, cirurgias adicionais, perda sanguínea e óbito em 30 dias).

### Responsabilidades da equipe

Os dados foram coletados a partir do atendimento de rotina de cada instituição e ficaram acessíveis on-line para todos os membros do grupo participante do projeto. A cada 2 meses, esses membros fizeram reuniões presenciais para discutir os dados parciais e, ao final do período estabelecido, houve uma reunião final para discutir os resultados completos e confeccionar o manuscrito.

### Análise estatística

Os dados contínuos foram expressos como média e desvio-padrão, e comparados por meio do teste *t* de Student para amostras não pareadas. Os dados categóricos foram comparados utilizando o teste exato de Fisher e os cálculos dos intervalos de confiança foram feitos. O nível de significância adotado foi de 5%. Foi utilizado o programa Epi-Info 7 para as análises.

## RESULTADOS

No período entre 2012 e 2022, um total de 951 casos consecutivos foram registrados, dos quais 152 eram mulheres (15,9%) e 799 eram homens (84,0%). Durante a pandemia de covid-19, a coleta e o registro de dados foram interrompidos. Todos os casos foram acompanhados por pelo menos 30 dias. Os dados demográficos estão apresentados na Tabela 1. Na Tabela 2, estão colocados os fatores de risco. A Tabela 3 mostra os dados anatômicos. Na Tabela 4, os dados cirúrgicos e complicações são apresentados.

**Tabela 1:** Dados demográficos dos pacientes registrados, sintomas e etiologia, de acordo com o sexo. Frequência em porcentagem.

	Característica	Feminino	Masculino	Valor de p
Dados demográficos	Idade média (anos)	70,5	71,5	0,1279
	Sexo	15,98%	84,02%	< 0,05^*^
	Etnia branco	80,26%	87,36%	0,5214
Sintomas	Assintomático	57,89%	65,83%	0,06075
Etiologia	Degenerativo	93,42%	95,11%	0,385435
	Inflamatório	0,00%	0,26%	-

* diferença significante.

**Tabela 2:** Fatores de risco dos pacientes registrados, de acordo com o sexo. Frequência em porcentagem.

Fator de risco	Feminino n = 152	Masculino n = 799	Valor de p
Diabetes	19,7%	19,7%	0,991425
Hipertensão	90,7%	44.4%	0,007463^*^
Dislipidemia	45,4%	33,1%	0,003793*
Tabagismo	51,9%	65,3%	0,001748*
Insuficiência renal	7,23%	7,63%	0,855092
Doença arterial coronariana	25,0%	20,1%	0,177873
Doença cérebro-vascular	7,23%	5,9%	0,52242

* diferença significante.

M = sexo masculino; F = sexo feminino.

**Tabela 3:** Aspectos anatômicos dos AAA, de acordo com o sexo. Frequência em porcentagem.

Tipo	Feminino	Masculino	Valor de p
Fusiforme	95,39%	89,86%	0,03066^*^
Sacular	3,94%	8,26%	0,065407
Indefinido	0,00%	1,25%	-
Diâmetro médio (cm)	5,960	6,496	0,0056*
Colo normal (> 15mm)	86,18%	81,22%	0,144599
Colo cônico	7,23%	5,75%	0,481166
Colo proximal curto	11,18%	15,26%	0,191312
Colo proximal angulado	11,84%	8,76%	0,229527
Colo com trombo	5,92%	4,75%	0,543545
Colo calcificado	8,55%	5,38%	0,127967
Colo distal < 15 mm	0,65%	0,12%	0,188789
Artérias ilíacas preservadas	89,40%	73,58%	0,00003*

* diferença significante.

**Tabela 4:** Complicações cirúrgicas dos AAA, de acordo com o sexo. Frequência em porcentagem.

Complicação	Feminino	Masculino	Valor de p
Conversão	0,65%	1,00%	0,688597
Estenoses	3,28%	3,75%	0,780068
Obstruções	15,2%	13,51%	0,017396^*^
Sem vazamentos	81,57%	81,72%	0,965447
Vazamento tipo I	3,94%	6,38%	0,246235
Vazamento tipo II	4,60%	9,13%	0,065072
Vazamento tipo III	0,0%	0,0%	-
Vazamento tipo IV	1,97%	2,13%	0,700064
Perda sanguínea	168,84 ml	321,40 ml	p < 0,05*
Permanência UTI (horas)			p = 0,1539
Óbito	4,60%	4,88%	0,884482

* diferença significante.

## DISCUSSÃO

Os autores desconhecem estudos multicêntricos prospectivos de registro de casos de AAA no Brasil e na América Latina. Esse registro foi criado e mantido com recursos próprios por um grupo de professores e cirurgiões vasculares de universidades públicas do interior de São Paulo (que fazem parte do RHEUNI). Isso requereu um sistema organizado de coleta de informações através de sistema computacional on-line, com servidor dedicado, confiável e dinâmico, com ferramentas de cálculos estatísticos e acessível para todos investigadores do projeto^13^ . No entanto, os dados dos pacientes foram sigilosos e confidenciais, e não houve nenhuma forma de identificação dos mesmos. Periodicamente, foram feitas reuniões da equipe para adjudicação dos dados coletados e análises parciais. Embora tenha limitações, a importância do registro de casos reside em avaliar resultados específicos de determinada doença da população^14^ .

Os registros internacionais prévios de AAA mostraram alguns indícios de comportamento diferencial de acordo com o gênero^15,16^ .

No estudo de Deery et al.^15^ , baseado em levantamento de dados do Medicare, de 6.611 pacientes (19% mulheres), 87% foram operados de AAA por meio de EVAR (83% das mulheres e 88% dos homens). As mulheres eram mais velhas (76 *versus* 73 anos), tinham aneurismas menores (5,4 *versus* 5,5 cm, p < 0,001) e tinham maior prevalência de doença pulmonar obstrutiva crônica (22% *versus* 17%, p = 0,001). Além disso, o tempo operatório das mulheres foi mais longo, e essas tiveram problemas renais e vasculares periféricos associados. A mortalidade foi maior nas mulheres (*odds ratio* [OR], 1,7; intervalo de confiança de 95%, 1,1-2,6; p = 0,02), assim como as complicações (OR, 1.4; IC, 1,1-1,7; p < 0,01).

O registro compulsório holandês (Dutch Surgical Aneurysm Audit [DSAA])^16^ incluiu 1.561 aneurismas rotos e 7.063 eletivos (13,7% mulheres). As mulheres eram mais velhas, tinham diâmetros aórticos significativamente menores no momento da ruptura e tiveram maior mortalidade de 30 dias após reparo de emergência. O reparo aberto foi associado a uma mortalidade duas vezes maior nas mulheres.

No registro sueco envolvendo 32.393 casos de AAA não rotos (diâmetro aórtico > 3,0 cm), cerca de 20.000 (60%) não foram tratados^6^ . A proporção de homens e mulheres foi similar, mas a frequência de ruptura foi maior nas mulheres em 5 anos (9,7% nas mulheres *versus* 6,9% nos homens, p < 0,001). Após 5 anos, 56,5% das mulheres e 50,4 dos homens morreram (p < 0,001). A ruptura foi a terceira causa mais frequente de morte (11,9% nas mulheres e 8,7% nos homens; p < 0,001). Os autores concluíram que se deveria estabelecer um programa de vigilância melhor para as mulheres com AAA.

No registro de 9.675 cirurgias não aderentes ao guia de práticas clínicas da Society for Vascular Surgery, os resultados de mortalidade e reintervenção foram piores nas mulheres^17^ .

Além dos registros, quatro revisões sistemáticas recentes mostraram que as mulheres com AAA, operadas por via aberta ou endovascular, tiveram maior mortalidade^18-20^ , maior frequência de aneurismas rotos^18^ , de complicações pós-operatórias^20^ e de transfusões, além de complicações pulmonares e intestinais^19^ . Na revisão sistemática de Patel et al.^8^ , observou-se grande disparidade no recrutamento e desfechos relacionados com sexo e etnia nos trabalhos relatando resultados do EVAR, embora os registros prévios já mostrassem indícios dessa diferença. Exclusivamente em cirurgia endovascular do AAA^21,22^ , a mortalidade nas mulheres foi mais frequente, assim como as reintervenções^21^ e anatomia hostil^22^ .

Em função dessas diferenças, surgiram propostas de se operar os AAA de mulheres com diâmetros menores que os estabelecidos para os homens^2,3,23-27^ .

Na presente casuística, a frequência de AAA foi menor em mulheres e as idades foram similares em ambos sexos. A etnia branca predominou igualmente entre mulheres e homens. A apresentação clínica, etiologia, tipo de colos proximal e distal, e colos complicados foram similares entre os sexos (Tabelas 1 e 3). A morfologia fusiforme foi mais frequente nas mulheres e as artérias ilíacas estavam significativamente mais preservadas nas mulheres (Tabela 3). Porém, as taxas de obstrução foram significativamente mais frequentes nas mulheres, possivelmente por conta da relação calibre dos dispositivos calibrosos/artérias menores nas mulheres. Esses achados corroboram parcialmente as conclusões dos registros prévios de AAA comparando homens e mulheres^8,15,16,28^ , apontando alguns desfechos piores no sexo feminino. Diferentemente de vários outros estudos, a mortalidade operatória na presente casuística foi similar entre ambos os sexos.

Deve-se salientar algumas limitações do presente estudo. A significativa menor frequência de casos do sexo feminino nesta casuística prejudicou as comparações, impedindo extrapolações para o universo de casos. Além disso, as indicações cirúrgicas e critérios ficaram ao escrutínio de cada equipe isoladamente, o que pode ter ocasionado um viés de seleção. Embora os serviços participantes fossem todos universitários e cientes das diretrizes, uma disparidade de condutas pode ter ocorrido. Finalmente, o seguimento dos casos foi feito em prazo curto (30 dias), o que impediu a avaliação de morbimortalidade de longo prazo. As vantagens deste estudo foram os registros prospectivos e consecutivos, em amostra de população brasileira atendida no Sistema Único de Saúde (SUS), com dados baseados em protocolo único para todos participantes, o que fez diminuir as perdas de dados, como costuma ocorrer em estudos retrospectivos.

Em conclusão, apesar do diâmetro menor e maior preservação das artérias ilíacas nas mulheres, as complicações obstrutivas nestas foram maiores com o tratamento endovascular. Para reduzir as complicações nas mulheres, a literatura sugere um perfil de endopróteses menor, com maior flexibilidade e implante cuidadoso.
